# Predictors of decision ambivalence and the differences between actual living liver donors and potential living liver donors

**DOI:** 10.1371/journal.pone.0175672

**Published:** 2017-05-17

**Authors:** Li-Chueh Weng, Hsiu-Li Huang, Hsiu-Hsin Tsai, Wei-Chen Lee

**Affiliations:** 1 School of Nursing, College of Medicine, Chang Gung University, Taoyuan, Taiwan; 2 Department of General Surgery, Chang Gung Medical Foundation-Linkuo, Taoyuan, Taiwan; 3 Department of Long-Term Care, National Taipei University of Nursing and Health Sciences, Taipei, Taiwan; 4 Department of Psychiatry, Chang Gung Medical Foundation-Linkuo, Taoyuan, Taiwan; University of Toledo, UNITED STATES

## Abstract

**Background:**

The decision to become a living liver donor is a stressful event. Ambivalence in decision making may result in psychological distress. Thus, the purpose of this study was to provide a description of the ambivalence of potential living liver donors, to examine the predictors of ambivalence, and to compare the ambivalence of potential living liver donors with that of actual living liver donors.

**Methods:**

This descriptive and correlational study was conducted in a medical center from August 2013 to December 2015. Self-reported questionnaires were used to collect data. A total of 263 potential living liver donors who were assessed for donation to their parents were included in this study.

**Results:**

The mean age of the total sample was 30.7 years (SD = 6.39, range = 20–47), and males comprised 53.6% of the sample. The majority of the potential donors had a college education (70.8%) and were single (63.5%). Of the total sample, the mean score for ambivalence was 4.27 (SD = 1.87, range = 0–7). Multivariate analysis revealed that the Mental Component Summary (MCS) of quality of life (*β* = -0.24, *p* < 0.01), family support (*β* = -0.17, *p* = 0.007), and intimacy (*β* = -0.13, *p* = 0.04) were significant protective predictors of ambivalence. Actual living liver donors had significantly lower ambivalence (3.82 versus 4.60), higher intimacy with recipients (3.55 versus 3.34), higher MCS (45.26 versus 42.80), and higher family support (34.39 versus 29.79) than did the remaining potential living liver donors.

**Conclusion:**

Ambivalence is common in potential living liver donors. The MCS of quality of life, family support, and intimacy were protective predictors in terms of ambivalence. Future research should explore other factors and design interventions targeted toward reducing ambivalence, promoting family support, and enhancing the mental dimensions of quality of life in potential living liver donors.

## Introduction

Living donor liver transplantation (LDLT) was developed in the 1980s to overcome the demands of liver transplantation and the shortage of deceased donor organs [1]. Although the rate of LDLT has been decreasing in the United States, the rate of LDLT has been increasing in Asian countries [2]. Between 1989 and 2001, the estimated rate of LDLT was 37% in Taiwan, 99% in Japan, and 66% in South Korea [3]. According to a more recent report, in 2015, the rate of LDLT was 83% in Taiwan [4]. In general, the outcomes of LDLT are similar to those of deceased liver transplantation [5]. Nevertheless, potential harm to a healthy donor’s health remains an ethical consideration [5–6]. Potential living liver donors should undergo a rigorous assessment and evaluation process to ensure their voluntary donation and to limit or prevent the negative consequence of donation [7–8]. In addition, living liver donors’ physical well-being as well as psychological well-being need to be protected throughout the process [9–11].

Research has shown that, even when donors want to save the life of the recipient and the decision to donate came easily, without coercion, donors may experience anxiety and other psychological distress during the assessment period [12–15]. Decision ambivalence, which is the coexistence of inconsistent or opposing perceptions [16], is a common feeling among potential living donors during the assessment process, with a prevalence rate of approximately 33.8% [17–20]. Ambivalence about living donor donation involves a variety of cognitions and emotional response, including worry, uncertainty, doubt, and reluctance. The phenomenon of ambivalence concerns the extent of the individual’s conflicting feelings [21–22]. Research also has shown that living liver donors who experienced more conflict or more pressure related to decision making are likely to experience higher psychological distress and more physical complaints after donation surgery [10, 23]. Notably, a supportive relationship among family members may lessen decision ambivalence [24]. Other factors, such as the donors’ physical and psychological status, the quality of the relationship between donors and recipients, and perceptions of family conflict, may contribute to decision ambivalence and warrant further investigation.

Understanding the extent of ambivalence and its associated factors is important for the transplant team as well as for the living donor advocacy team. Predictors of ambivalence should be included in the assessment and evaluation protocol and used to identify risk. Moreover, an understanding of the association between certain factors and ambivalence may provide a theoretical framework for establishing evidence-based intervention. Nevertheless, studies that explore ambivalence and its predictors are scarce. Thus, the purpose of this study was to provide a description of the ambivalence of potential living liver donors, to examine the predictors of ambivalence, and to compare the ambivalence of potential living liver donors and with that of actual living liver donors.

## Materials and methods

### Sample

The study used a descriptive and correlational design. The study site was a medical center in northern Taiwan. Based on government regulations, legislation in regard to living liver transplantation pertains to only the recipient’s spouse and relatives within fifth-degree consanguinity. The potential living liver donor evaluation protocol in this center involved a nurse coordinator who reviews the procedures of the evaluation process, provides the information related to LDLT (e.g., indications, adverse effects), and arranges for donor candidates to receive further evaluation, including laboratory/radiological tests and consultation with a psychiatrist and social worker. Candidates who choose to become donors receive further consultation with a surgical physician before making the final decision. The data are then sent to an ethics committee for review and then to a government-approval institute for authorization to proceed with the operation.

The inclusion criteria for this study for potential living liver donors (PLDs) was age 20 years or older, able to communicate in Mandarin, and able to provide written consent to participate. Participants were informed of the purpose of the research, and written consent was obtained. Participants were assured of their right of refusal to participate or to withdraw from the study at any stage, and anonymity was assured. Participants’ names were removed from the data, and a numerical code was used in place of their names. This study was approved by the Chang Gung Medical Foundation Institutional Review Board (Approval No. CGMH 102-1974B).

During the period of recruitment (August 2013 to December 2015), 385 PLDs were approached. Of these, 367 met the inclusion criteria (16 were excluded due to their being under 20 years old, and 2 could not speak Mandarin), but 28 declined to participate (5 were not interested, and 23 indicated a busy schedule), resulting in 339 candidates who were assessed for the 219 recipients included in this study. There were no significant differences in age or proportion of gender between participants and non-participants.

Of the 339 PLDs, 27 were assessed for their spouse, 26 for siblings, 23 for other relatives, and 263 for their parents. To ensure homogeneity of the decision experience, we report on only the 263 PLDs who were assessed for their parents. After the assessment, the PLDs were classified into two groups: the accepted and actual living liver donors (ALDs, *n* = 112 [42.6%]) and the rejected (remaining PLD, *n* = 151 [57.4%]).

### Variable measurement

#### Basic data

PLD characteristics included age, gender, educational level, marital status, and religion (yes or no). Recipient characteristics included current health condition (critical or stable) and alcohol-related liver cirrhosis (yes or no). Potential donors self-reported their intimacy level with recipients (0–4; 0 = not close at all, 1 = not very close, 2 = somewhat close, 3 = close, and 4 = very close).

#### Ambivalence

The Ambivalence subscale of the Donor Attitude Scale, developed by Simmons, Marine, and Simmons [21], was used to measure the extent of ambivalence of donors [20]. This subscale contains seven items. The item, “Did you know right away you would definitely do it, or did you think it over?” is answered on a 2-point scale: A response of 0 indicates that the decision was made instantly, and 1 indicates that the decision required thought. The other six items are answered on a 4-point Likert scale. The score is calculated per the following: “How hard was the decision to make?” Responses include that it felt “very hard,” “somewhat hard,” “a little hard,” or “not at all hard.” If the answer is “very,” “somewhat,” or “a little,” then the score is 1; if the answer is “not at all hard,” then the score is 0 because this answer indicates that there is no ambivalence. The range of scores on the Ambivalence subscale is 0–7, with a higher score’s indicating a higher degree of ambivalence. Because we thought that PLDs would experience different degrees of ambivalence, we used the summary score to indicate the extent of ambivalence of each potential living donor in this study. The Chinese version of the Ambivalence subscale was developed through translation, and permission for its use was obtained [25]. This scale also has been used with living liver donor candidates [24]. Validity and reliability were confirmed in previous studies [24]. Cronbach’s *α* was 0.71 in this study, which indicates acceptable internal consistency reliability.

#### Physical and psychological well-being

A generic health-related quality of life scale, the Medical Outcome Survey (MOS SF-36), Taiwanese version, was used to measure physical and psychological well-being [26]. The 36-item instrument consists of eight aggregate scales: Physical Functioning (PF), Role Physical (RP), Bodily Pain (BP), Vitality (VT), General Perception of Health (GH), Social Functioning (SF), Role Emotional (RE), and Mental Health (MH). This scale is commonly used when studying transplantation and has good reliability and validity [27]. The score of the eight scales are calculated into two dimensions as the Physical Component Summary (PCS) to indicate physical well-being and the Mental Component Summary (MCS) to indicate psychological well-being. In this study, PCS and MCS scores were used for statistical analysis.

#### Family support

Family support was measured by a self-report scale with 16 items, answered on a 4-point Likert scale (with a range of 0 to 3) as a means to assess emotional support (as emotional interaction, making someone feel love and joy), valuable support (as providing feedback, affirming the support of one’s values), instrumental support (as providing practical assistance, e.g., household help), and information support (as providing teaching, counseling, and information) [28]. A higher score indicates higher family support. Reliability and validity were tested and confirmed [28]. Cronbach’s *α* was 0.94, which indicates acceptable internal consistency.

#### Family conflict

The nine-item Family Conflict subscale of the Family Environment Scale, developed by Moos, was used to assess the amount of openly expressed anger and conflict among family members [29]. Items include, “We fight a lot in our family.” The items are answered as *true* (1) or *false* (0), with a possible score of 0 to 9. Higher scores indicate that the amount of expressed anger and conflict is high among family members. The internal consistency reliability Cronbach’s *α* was 0.69, which indicates acceptable internal consistency.

### Study process

During the potential donor evaluative clinical visits, a trained research assistant approached and invited the potential donors to participate in the study. Participants were informed of the purpose of the research, and written consent was obtained. Participants were assured of their right of refusal to participate or to withdraw from the study at any stage, and anonymity was assured. Participants’ names were removed from the data, and a numerical code was used in place of their names. This study was approved by the Institutional Research Ethics Committee (Approval No. CGMH 102-1974B).

### Statistical analyses

Data were entered into SPSS software, Version 22.0, for statistical analysis (IBM, New York, NY). Descriptive data were used for estimates of central tendency (mean, median) and spread (standard deviation, range) for continuous data, and frequencies and percentages were used for categorical data. Pearson correlations were used to determine the relationship between continuous variables (age, intimacy level, PCS, MCS, perceived family support, and family conflict) and ambivalence. Independent-sample *t*-tests and one-way ANOVAs were used to compare the differences in ambivalence among categorical variables (gender, religion, marital status, education, recipients’ condition, and alcohol-related liver cirrhosis). Significant variables in the univariate analyses were then included in a multiple linear regression model (with a forward method) to determine the predictors of ambivalence [30]. The statistical assumption of normal distribution was determined prior to the regression analysis. In addition, independent-sample *t*-tests were used to compare ambivalence, intimacy level, PCS, MCS, perceived family support, and family conflict between the remaining PLD and ALD groups. The significance level was set at 0.05.

## Results

### Basic data

The mean age of the total sample was 30.7 years (SD = 6.39, range = 20–47), and males comprised 53.6%. Most PLDs had a college education (70.8%) and were single (63.5%). In addition, 61.6% of recipients were in stable health, 19.4% had alcohol-related liver cirrhosis, and 42.6% of PLDs actually donated (Table 1).

**Table 1:** Basic data of study sample (*N* = 263).

Variable	*n*	*%*	Mean	SD
Age (Range: 20–47)			30.70	6.39
Gender				
Female	122	46.4		
Male	141	53.6		
Religion (*n* = 260)				
Yes	142	54.6		
No	118	45.4		
Marital Status				
Married	80	30.4		
Single	167	63.5		
Divorced	16	6.1		
Education				
Less than high school	9	3.4		
High school	68	25.9		
College	186	70.7		
Sole candidate				
Yes	74	28.1		
No	189	71.9		
Recipient condition				
Critical and hospitalized	101	38.4		
Stable	162	61.6		
Alcoholic-related liver disease				
Yes	51	19.4		
No	212	80.6		
Donated				
Yes	112	42.6		
No	151	57.4		

### Ambivalence

The mean score of ambivalence was 4.27 (SD = 1.87, range = 0–7), which indicated that the extent of ambivalence was moderate. The mean score of intimacy level, PCS, MCS, family support, and family conflict are shown in Table 2. The raw data was available in S1 File.

**Table 2:** Scores for ambivalence, intimacy, PCS, MCS, family support, and family conflict (*N* = 263).

Variable	Mean	SD	Median	Range
Ambivalence	4.27	1.87	5	0–7
Intimacy	3.43	0.74	4	0–4
PCS	56.97	5.96	58	27–71
MCS	43.86	9.43	45	10–60
Family support	31.74	12.45	32	0–48
Family conflict	2.25	1.97	2	0–7

Note: PCS = Physical Component Summary, MCS = Mental Component Summary

### Predictors of ambivalence

The univariate analysis showed that ambivalence was significantly negatively correlated with intimacy (*r* = -0.23, *p* < 0.01), MCS (*r* = -0.29, *p* < 0.01), and family support (*r* = -0.25, *p* < 0.01) and positive correlated with family conflict (*r* = 0.15, *p* = 0.02) (Table 3).

**Table 3:** Correlations of age, intimacy, PCS, MCS, family support, and family conflict with ambivalence (*N* = 263).

Variable	Ambivalence	
	*r*	p-value
Age	0.06	0.37
Intimacy	-0.23	<0.001**
PCS	-0.06	0.37
MCS	-0.29	<0.001**
Family support	-0.25	<0.001**
Family conflict	0.15	0.02*

Note: PCS = Physical Component Summary, MCS = Mental Component Summary

*p < 0.05,

**p < 0.01

Degree of ambivalence did not significantly differ among basic characteristics, including age, gender, religion, education level, and marital status, for either PLDs or their recipients (Table 4).

**Table 4:** Comparison of the degree of ambivalence among different characteristics (*N* = 263).

Characteristic	Mean	SD	*t* or *F*	*p-*value
Gender				
Female	4.31	1.93	0.33	0.74
Male	4.23	1.81		
Religion				
No	4.21	1.96	-0.47	0.63
Yes	4.32	1.80		
Marital status				
Single	4.28	1.83	0.25	0.77
Married	4.30	1.89		
Divorced	3.93	2.25		
Education				
Less than high school	5.22	1.20	1.22	0.29
High school	4.24	1.85		
College	4.23	1.89		
Sole candidate				
Yes	4.18	1.64	0.51	0.61
No	4.30	1.96		
Recipient status				
Stable	4.27	1.90	0.01	0.99
Critical	4.27	1.82		
Alcohol-related				
No	4.34	1.82	1.28	0.20
Yes	3.96	2.04		

Multivariate analysis revealed that MCS (*β* = -0.24, *p* < 0.01), family support (*β* = -0.17, *p* = 0.007) and intimacy (*β* = -0.13, *p* = 0.04)) were the significant protective predictors of the degree of ambivalence. The explained variance (adjusted *R*^*2*^) was 13% (*F* = 13.67, *p* < 0.001) (Table 5).

**Table 5:** Predictors of ambivalence: Multiple linear regression analysis (*N* = 263).

Variable	*B*	*SE*	*β*	*p-value*	95% CI for *B*	
					Lower bound	Upper bound
MCS	-0.05	0.01	-0.24	0.001**	-0.07	-0.02
Family support	-0.03	0.01	-0.17	0.007**	-0.05	-0.07
Intimacy	-0.33	0.16	-0.13	0.040*	-0.65	-0.02

Note: MCS = Mental Component Summary,

*p < 0.05,

**p < 0.01

Explained variance (adjusted *R*^*2*^) = 0.13 (13%), *F* = 13.67, *p* < 0.001

### Comparison between remained PLDs and ALDs

Independent-sample *t*-tests revealed that ALDs had significantly lower ambivalence (3.82 versus 4.60), higher intimacy with recipients (3.55 versus 3.34), higher MCS (45.26 versus 42.80), and higher family support (34.39 versus 29.79) than did the remaining PLDs. PLDs who were sole candidates (*χ2* = 21.19, *p* < 0.001), with recipients in stable condition, tended to be actual donors (*χ2* = 4.76, *p* < 0.03). PCS and family conflict did not show a significant difference between two groups. Other basic characteristics were not significantly different between the remaining PLDs and ALDs (Table 6).

**Table 6:** Comparison of ambivalence, intimacy, PCS, MCS, family support, family conflict, and basic characteristics between remaining PLDs and ALDs (*N* = 263).

Variable	Remaining PLDs (*n* = 151)	ALDs (*n* = 112)	*t*	*p*-value
	Mean ± SD	Mean ± SD		
Ambivalence	4.60 ± 1.83	3.82 ± 1.83	3.38	0.001*
Intimacy	3.34 ± 0.78	3.55 ± 0.68	-2.30	0.022*
PCS	58.84 ± 6.25	57.14 ± 5.56	-0.40	0.680
MCS	42.80 ± 9.68	45.26 ± 8.92	-2.08	0.038*
Family support	29.79 ± 12.83	34.39 ± 11.47	-2.95	0.003*
Family conflict	2.34 ± 2.02	2.12 ± 1.89	0.87	0.380
Age	30.7 ± 6.05	30.8 ± 6.85	-0.21	0.840
	*n (%)*	*n (%)*	*χ2*	*p*
Gender				
Female	69 (45.7)	53 (47.3)	0.02	0.809
Male	82 (54.3)	59 (52.7)		
Religion				
No	66 (44.6)	52 (46.4)	0.03	0.860
Yes	82 (55.4)	62 (53.6)		
Marital status				
Single	97 (64.2)	70 (62.5)	4.99	0.080
Married	49 (32.5)	31 (27.7)		
Divorced	5 (3.3)	11 (9.8)		
Education				
Below high	6 (4.0)	3 (2.7)	2.23	0.330
High school	34 (22.5)	34 (30.3)		
College	111 (73.5)	75 (67.0)		
Sole candidate				
No	126 (83.4)	63 (56.3)	21.19	<0.001**
Yes	25 (16.6)	49 (43.8)		
Recipient status				
Stable	84 (55.6)	78 (69.6)	4.76	0.030*
Critical	67 (44.4)	34 (30.4)		
Alcohol-related				
No	119 (78.8)	93 (83.0)	0.49	0.480
Yes	32 (21.2)	19 (17.0)		

Note: PLDs = potential living liver donors; ALDs = actual living liver donors, PCS = Physical Component Summary, MCS = Mental Component Summary,

*p < 0.05,

**p < 0.01

## Discussion

In this study, the degree of ambivalence was higher than that of previous studies that used the same ambivalence scale; DiMartini et al. [20] found a mean score 2.7, and Lai et al. [24] found a mean score 3.14. In traditional Eastern culture, there is an emphasis on moral obligation and family order [31–32]. Thus, PLDs may feel obligated to risk their mental and physical health, which may be related to higher ambivalence, to save the recipient’s life and preserve the family order. Indeed, donor candidates must make a decision that will have a major impact on the lives and well-being of their family, including the recipients and other family members [19]. All of the donor candidates included in this study were adults who were serving as donors for adult liver transplant recipients. A study that explored the influence of kinship on donors’ mental health found that adult children who donated for their parents showed the highest mental stress and conflict, as compared to that of other relatives [33]. In Japan, Muto found that adult donors had several concerns, including care burden, maintaining employment, and change in family relationships [34]. Higher ambivalence may indicate that donors have given considerable thought to the pros and cons of donation. Healthcare professionals need to provide suitable information as well as to promote open discussion and communication among candidates, recipients, and their family members [20].

Multivariate analysis revealed the significant factors associated with ambivalence. MCS, in particular, is a protective factor in regard to ambivalence. The MCS dimension of quality of life includes psychological strength. This strength protects the potential donor from the stress of decision making and, in turn, reduces ambivalence. In a study of 165 living liver donor candidates, Uehara et al. found that candidates’ psychological traits (higher trait anxiety and higher alexithymia score) could lead to a “postponement decision,” and, thus, there is a need to provide appropriate psychological support [35]. Our results suggest that the quality of life of the PLD should be included in the assessment and evaluation protocol.

The quality of the donor-recipient relationship, specifically their intimacy, was a protective factor in regard to ambivalence. In Taiwan, in a study of 100 living liver donor candidates, Lai et al. found that an intimate relationship between a PLD and recipient could reduce ambivalence in cases in which there are psychosocial concerns about donation [24]. The quality of the donor-recipient relationships was a key factor in the willingness to donate one’s organ [36–37]. Intimacy or a close relationship between a donor and recipient indicates that they spend time with each other and have a strong emotional bond. In such cases, the decision to become a donor may involve no ambivalence. In contrast, a relationship with little or no intimacy may be indicative of pre-existing anxiety or anger toward the recipient, but the social expectation or moral obligation may cause the donor to be filled with ambivalence regarding the decision [34, 37].

Most countries’ legislation in regard to living liver transplantation pertains to the recipient’s spouse and relatives with consanguinity, which means that decision making in living liver donation is regarded as a family issue [34]. Family is an important resource for individual growth and development in terms of physical, psychological, and social aspects [38–39]. Our results show that family support had a protective effect in regard to decision ambivalence and were similar to those of Lai et al., who found that high family support can reduce the impact of psychosocial concerns on the extent of ambivalence in PLDs [24]. Erim et al. studied the effect of social support on 71 donor candidates who were evaluated for adult or pediatric liver transplantation. The results showed that candidates who perceive higher support will experience lower depression. The authors concluded that family support can help a donor to cope with stress of donation [40]. Indeed, in this study, family conflict exacerbated ambivalence. When faced with conflict, a PLD’s decision may involve more ambivalence. Fortunately, as seen in the multivariate analysis, the impact of family conflict was attenuated by other protective factors. During the assessment stage, healthcare professionals should be sensitive to detecting family conflict and to promoting family support.

Our study also provided a comparison between PLDs and ALDs. The results showed that ALDs experience significantly lower ambivalence than do PLDs. Potential donors who express obvious ambivalence when making their decision may be experiencing coercion or be unwilling to donate. Research has suggested that, in terms of the negative impact of ambivalence on recovery and health status, overtly ambivalent potential donors should be excluded from being actual donors [18, 36, 41]. It may be the case that ambivalence among donors may indicate that they thought through the risk and benefits and that donors with no ambivalence may not be aware of the risks and negative consequences of donation or may be overly optimistic [19, 42]. No study, however, determined how much PLD ambivalence is reasonable and acceptable. In this study, the mean score of ambivalence for actual donors was 3.8. We suggest that a score of 4 or more is an indicator that an intervention should be provided by healthcare professionals. This suggestion warrants further research. Simpson et al. suggested that ambivalence should not be the sole reason for disqualifying donor candidates and suggested that it is appropriate to consider other psychological indicators [19]. Our study also revealed that ALDs had a better mental dimension of quality of life than did PLDs during assessment stage. This is similar to the findings of Schulz et al., who reported that ALDs had a higher, but not statistically significantly different, mental dimension of quality of life than did PLDs before donation [43]. ALDs also had more intimacy with recipients and higher family support than did PLDs. Thus, these factors may be considered indicators to include in an assessment and evaluation protocol. In addition to the use of interviews for assessment or consultation, well-structured scales and questionnaires, with good reliability and validity, are valuable tools.

This study had at least one limitation. The study used a cross-sectional design, and, thus, causality between factors and ambivalence can be inferred only with caution. Nevertheless, the findings of the current study help us to understand the decision ambivalence of PLDs before donation. This study did not explore post-donation ambivalence. Comparing pre- and post-donation ambivalence and determining the reason for any changes may be useful for understanding the impact of ambivalence. Future research should use prospective and longitudinal follow-up designs. In the regression model of this study, the explained variance was only 13%, which indicated that there were more predictors related to ambivalence that warrant investigation. We suggest that future research examine the effect of family structure and function, donors’ individual coping abilities, and strategies related to the lessening of ambivalence.

## Conclusions

In sum, the extent of decision ambivalence of PLDs was moderate. The mental dimension of quality of life, donor-recipient intimacy, and family support were significant predictors of the decision ambivalence of PLDs. ALDs had lower ambivalence, better intimacy with recipients, better mental quality of life, and more family support than did PLDs. Decision ambivalence is an important concern that needs to be assessed and managed carefully. To reduce decision ambivalence, healthcare professionals should focus on the donor-recipient relationship, social support, and quality of life when providing an intervention. In addition, assessment of ambivalence and other psychosocial factors as well as the donor-recipient relationship, social support, and quality of life may help to determine the suitability of candidate’s selection.

## Supporting information

S1 File Dataset. (XLSX)
